# Understanding the Molecular Mechanism and Structure-Function Relationship of the Toxicity of PLA_2_ and K49 Homologs in Snake Venom

**DOI:** 10.1155/2013/243047

**Published:** 2013-01-31

**Authors:** Luis Alberto Ponce-Soto, Laura Leiva, Elen Cristina Teizem Landucci

**Affiliations:** ^1^Department of Biochemistry, Institute of Biology, State University of Campinas (UNICAMP), CP 6109, 13083-970 Campinas, SP, Brazil; ^2^Laboratorio de Química Biológica, Departamento de Bioquímica, Facultad de Ciencias Exactas y Naturales y Agrimensura, Universidad Nacional del Nordeste (UNNE), Avenida Libertad 5470, Campus Universitario, CP 3400 Corrientes, Argentina; ^3^Laboratório de Inflamação, Departamento de Farmacologia, Faculdade de Ciências Médicas, Universidade Estadual de Campinas (UNICAMP), R. Tessália Vieira de Camargo, 126, Caixa Postal 6111, 13084-971 Campinas, SP, Brazil

The snake venom PLA_2_s, in association with the catalytic function that defines them, have developed a diverse set of biological functions which go beyond the purely digestive. Striking examples are the PLA_2_ that have evolved as toxins, either in conjunction with other proteins or by themselves, and in spite of having a similar structural geometry, they exert an amazing variety of pharmacological effects, which include myotoxic, neurotoxic, hemolytic, edematogenic, hyperalgesic, pro- and anti-inflammatory, hypotensive, anticoagulant, platelet-aggregation inhibitory, and cytotoxic. Furthermore, since the first studies reported by Maraganore et al. [1] to the present, in homologous K49 PLA_2_ from snake venom, have increased significantly. Despite its inability to bind Ca2 ion co-factor crucial for catalytic activity, it is capable of triggering toxic or pharmacological effects independent of PLA_2_ activity, thus opening a new understanding of the involvement of independent regions or domains of the catalytic site, postulated from Condrea et al. [2] and reinforced by Majunatha Kini and Evans [3]. The present work highlights some of the most relevant contributions in the study of venom PLA_2_s, including the PLA_2_ homólogous K49.

This special issue of contains nine papers describing structural and functional analysis of different PLA_2_ and PLA_2_ homólogous K49 from snakes. The review by C. V. Carregari and colleagues discusses the importance of the proinflammatory activity that characterized this new venom from *Bothriopsis bilineata* snake venom. The fact that Bbil-TX elicited a stronger inflammatory reaction argues in favor of a role of enzymatic phospholipid hydrolysis in this phenomenon, either through the direct release of arachidonic acid from plasma membranes or through activation of intracellular processes in target cells. I. G. Rodríguez et al. analyze, the PLA_2_s isolated from Panama *Bothrops asper* venoms (pMTX-I, II, III, and IV), which are able to induce myotoxic activity, inflammatory reaction, and mainly leukocyte migration to the muscle and induce J774A.1 macrophages activation to start phagocytic activity and superoxide production. J. Nelson et al. make an important contribution to the study of synergistic effects of secretory PLA_2_ from the venom of *Agkistrodon piscivorus piscivorus* with cancer chemotherapeutic agents. Healthy cells typically resist hydrolysis catalyzed by snake venom secretory PLA_2_. However, during various forms of programmed cell death, they become vulnerable to attack by the enzyme. This observation raises the question of whether the specificity of the enzyme for dying cells could be used as a strategy to eliminate tumor cells that have been intoxicated but not directly killed by chemotherapeutic agents. This work suggests that exposure of lymphoma cells to these drugs universally causes changes to the cell membrane that render it susceptible to enzymatic attack and that the snake venom enzyme is not only capable of clearing cell corpses but can aid in the demise of tumor cells that have initiated but not yet completed the death process. L. Wei et al. present a valuable contribution to the study of induction in the accumulation of mast cells, promutoxin, a new variant of PLA_2_ R49. The action of an R49 PLA_2_s, promutoxin from *Protobothrops mucrosquamatus* venom on mast cell accumulation, has not been previously examined. The promutoxin-induced mast cell accumulation was inhibited by cyproheptadine, terfenadine, and ginkgolide B, indicating that histamine and platelet activation factor (PAF) are likely to contribute to the mast cells accumulation. Preinjection of antibodies against adhesion molecules ICAM-1, CD18, CD11a, and L-selectin showed that ICAM-1, CD18, and CD11a are key adhesion molecules of promutoxin-induced mast cell accumulation. Promutoxin, as a novel member of minor subgroup of PLA_2_, is an enzymatically inactive enzyme. It induced mast cell accumulation via a PAF and histamine H1 receptor-dependent mechanism and through a CD11a/CD18 and ICAM-1 associated adhesion pathway. F. A. Marangoni et al. present an important contribution to the study structure function of a new PLA_2_ isolated from *Bothrops leucurus*. Kinetic and pharmacological studies illustrate a behavior similar to other PLA_2_ from snake venom Viperidae; however from the structural point of view, in relation to the few differences in its sequence, the contribution of each region or domain, as well as each amino acid, has been crucial in the understanding of toxic or pharmacological activities. 

S. Huancahuire-Vega et al. analyze the use of chemical modifications of a new PLA_2_ from *Porthidium hyoprora* snake venom in the study structure-function relationships. The results supported the hypothesis that both the catalytic sites as the hypothetical pharmacological sites are relevant to the pharmacological profile of PhTX-I. K. Giannotti et al. discuss an interesting study on the pathogenesis of the inflammatory process induced by a homologous K49 PLA_2_ from the venom of the snake *Bothrops asper*. The MT-II (PLA_2_ homologous K49) induces lipid droplet formation in macrophages that depends on distinct signaling pathways and the C-terminal region. MT-II directly activates murine macrophages to form LDs by a mechanism independent of enzymatic activity. This effect is related to the C-terminal loop of the MT-II molecule since a synthetic peptide corresponding to region 115–129 induced LD formation similarly to MT-II. Moreover, MT-II-induced LD formation is related to increased expression and recruitment of PLIN2 from its constitutive pools and regulated by distinct signaling pathways that include PKC, PI3K, ERK1/2, and iPLA_2_. In addition, MT-II induced synthesis and compartmentalization of PGE2 within LDs. Therefore, LDs may represent an important platform for the synthesis and accumulation of lipid mediators under MT-II stimulus, that takes place in the mechanisms whereby this PLA_2_ homologous K49 triggers inflammation. M. A. G. Heleno et al. conduct a major study on the structure-function basis of a new PLA_2_ from *Bothrops roedingerii*, indicating that their enzyme profiles as toxic or pharmacological show similar behavior to other PLA_2_. BrTX-I caused a neuromuscular blockade in biventer cervicis preparations in a similar way to other *Bothrops* species. BrTX-I induced myonecrosis and oedema-forming activity analyzed through injection of the purified BrTX-I in mice. Since BrTX-I exert a strong proinflammatory effect, the enzymatic phospholipids hydrolysis might be relevant for these phenomena, incrementing levels of IL-1, IL-6, and TNF*α*.

The best approach to understanding structure-function mechanisms of PLA_2_ from snake venoms will present a comprehensive view of toxinologists in different research fields, including biochemistry, biophysics, pharmacology, toxicology, and medicine. We hope that this special issue will encourage researchers to take on this challenge and increasingly elucidate structure-function behavior of PLA_2_ and K49 counterparts in snake venom.

*Luis Alberto Ponce-Soto* *Laura Leiva* *Elen Cristina Teizem Landucci*
